# Age and Sex Differences in the State and Relationships between Process and Product Assessments of Fundamental-Motor Skills in Five to Eight-Year-Olds: The ExAMIN Youth SA Study

**DOI:** 10.3390/ijerph19159565

**Published:** 2022-08-03

**Authors:** Anita E. Pienaar, Makama A. Monyeki, Dané Coetzee, Barry Gerber, Wilmarié du Plessis, Aletta M. du Plessis, Ruan Kruger

**Affiliations:** 1 Physical Activity, Sport and Recreation (PhASRec), Potchefstroom Campus, North-West University, Potchefstroom 2531, South Africa; andries.monyeki@nwu.ac.za (M.A.M.); dane.coetzee@nwu.ac.za (D.C.); barry.gerber@nwu.ac.za (B.G.); 20376138@nwu.ac.za (W.d.P.); 20268319@nwu.ac.za (A.M.d.P.); 2 Hypertension in Africa Research Team (HART), North-West University, Potchefstroom 2531, South Africa; ruan.kruger@nwu.ac.za; 3 MRC Research Unit for Hypertension and Cardiovascular Disease, North-West University, Potchefstroom 2531, South Africa

**Keywords:** age, fundamental motor skills, health, locomotor skills, object control skills, physical education, process, product, sex

## Abstract

Adequate development of Fundamental Motor Skills (FMS) at a young age benefit holistic development and positive health outcomes. This study determined age and sex developmental differences in the state and the relationships between process and product assessments of four fundamental-motor skills (FMS) in five to eight-year-olds. An availability sample of 636 children; 291 boys, 345 girls, mean age of 6.8 ± 0.97 years in the North West Province of South Africa participated in the study. Spearman rank order correlations analyzed relationships between assessments, while sex and age differences were examined using independent t-testing and one-way ANOVA. Age showed significant developmental trajectories in all FMS. Older children were found to be more at risk for not learning FMS to mastery, while unique developmental patterns were also established compared to international trends. Strong to moderate significant practical associations (*p* < 0.05) emerged between process and product assessments in catching (r = 0.79), jumping (r = 0.40) running (r = −0.33) and kicking (r = 0.20), while also confirming that the strength of the associations varies depending upon the skill type. Associations strengthened with increasing age, were higher in girls in all FMS, while associations between behavioral criteria in all FMS also differed between FMS and sexes. These strong associations, need to be taken into consideration during the development of FMS towards obtaining full mastery.

## 1. Introduction

Providing young children with the best possibilities to fully develop their fundamental-motor skills (FMS) is essential, and beneficial to a child’s holistic development [1,2,3]. Additionally, FMS are foundational skills for more advanced skills to be mastered and are therefore perceived as the early building blocks for health, sport, and lifelong participation in physical activities, while also contributing to scholastic success [4,5,6]. It is well established that children achieve movement proficiency through functional play and physical activity [7,8], but of public health, the concern is that active lifestyle patterns during the early development phase changed over the years to a more sedentary lifestyle pattern.

In general, FMS should be well developed, also known as reaching the mature phase of development, between the ages of six and seven years [8]. However, researchers indicate declining trends in FMS proficiency. A worldwide decline in children’s mastery of gross motor skills is reported over 15 to 20 years [9]. More recent studies confirmed a lowering trend of 30% in FMS proficiency over the past decades (CHILT project, [10,11]. In addition, the “Move it, Groove it” study on nine and ten-year-old Australian children [12], a study on six to eight-year-olds in New South Wales [13], and three- to ten-year-old Brazilian children also report declining trends [14]. Furthermore, researchers reported that as many as 78% within any given sample of United States (USA) preschoolers show developmental delays in their FMS, suggesting a secular decline in their FMS when compared to normative references from 20–30 years ago [15]. Similarly, a study on six-year-old Flemish pre-schoolers [16] reported a good mastery in only six of seventeen FMS. Studies undertaken in South Africa on three to six-year-olds [17,18], seven to nine-year-old girls [19], and nine to ten-year-olds [20] also confirmed lowering trends. Not surprisingly, researchers stress that the prevalence of FMS mastery among children needs improvement [13]. To support children in improving their FMS proficiency, an accurate understanding of what age-appropriate developing characteristics are to be expected from them, is therefore needed. 

When studying the level of FMS mastery, the influence of sex should always be considered. Literature reports sex differences relating to locomotor and object control skills, especially in skipping, kicking, and striking skills [14,16,18,20,21,22]. Equivocal findings are, however, reported for catching skills in different studies [16,18,20,23]. Studies also report that differences are more pronounced between boys and girls in object control skills than in locomotor skills [16,18,20,21,22]. 

Researchers [24] highlighted that the monitoring of motor competence through motor testing is essential for determining developmental status, identifying health-related risks, and developing recommendations based on motor test results. FMS is usually analyzed and described as a process (qualitative) or as a product (quantitative) [25]. A qualitative or process-oriented assessment is a more detailed assessment that judges the quality of performance criteria of the skill execution. In this regard, statistics reported on the qualitative mastering of running, catching, kicking, and jumping skills, indicate that all are still developing between five to eight years of age [26]. At the age of five, running skills showed the highest rate of mastery (54%), while catching skills were mastered at a much smaller percentage (15%). A large percentage of children have also not yet fully mastered catching at eight years of age [26]. Most existing studies which assessed children’s FMS development, however, used product measurements and mostly focused on the detection of deficits in this area. The use of both process and product assessments is, however, suggested to comprehensively capture levels of motor competence (MC) in human movement [27,28]. More insight can also be obtained into the changes in motor development through a combination of process and product assessments of development [29].

Only a few studies are reported where both process and product analyses; and their relationships with assessment criteria were investigated [17,28,30,31,32]. It is concluded [28] that there is currently neither a clear nor a comprehensive understanding of the relationship between process- and product-oriented assessments of FMS competence in children. In agreement, another researcher [32] is of the opinion that definite assumptions of the strength of relationships are still lacking in this area of research. 

This discussion highlights that more understanding is required about the development, but also the accurate assessment of fundamental-motor skills in young children to fully support them in optimizing their FMS. It is therefore important to identify the current FMS developmental profiles of boys and girls Due to the limited studies conducted to date, there is also a lack of clarity about the association between the process and product nature of the execution of FMS, and hence the information obtained from different forms of assessment of FMS. Up to date, very little is still known about the qualitative developmental characteristics of FMS in healthy South African children, especially in the age group between five and eight years, while the relationship between process and product characteristics of FMS is also not well-understood. This study aimed to determine the state of development of running, catching, kicking, and jumping by means of process and product assessments. A secondary purpose of this study is to examine the association between process and product outcomes of FMS in children and the information they provide, especially in SA children between the ages of five and eight. It is hypothesized that these children will be developmentally delayed compared to their peersand that performance of these skills, based on process and product assessment, will be significantly associated.

## 2. Materials and Methods

This study received ethical approval from the Health Research Ethical Committee of the Faculty of Health Sciences of the North-West University for both the ExAMIN Youth SA (NWU-00091-16-A1) and BC–IT studies (NWU-00025-17-A1). The study forms part of the combined Exercise, Arterial Modulation and Nutrition in Youth South Africa (ExAMIN Youth SA) and the Body Composition (BC) by Isotope Techniques (BC–IT) longitudinal study. Both the ExAMIN Youth SA and BC–IT studies’ samples were drawn from apparently healthy children from the same schools simultaneously [33]. The ExAMIN Youth SA study is an analytical, multidisciplinary, observational cohort study, designed to investigate the interplay between body composition, motor- and health-related fitness and physical activity and salivary biomarkers in 1103 purposefully selected children, aged five to nine years attending public primary schools in the North West Province, South Africa. Only the baseline data of this study that was gathered between 2017 and 2019 were used for the purpose of this study. The North West Province Department of Education, principals from the participating schools, the parents, and their participating children provided approval for the study. Children were recruited from 10 public schools to ensure a good distribution in terms of socio-economic background.

Children from ten urban schools with a quintile status of 3, 4, and 5 in two of the southern municipal areas namely JB Marks (Potchefstroom) and Matlosana (Klerksdorp) within the Kenneth Kaunda school district of the North West Province of South Africa participated in the study. A conservative calculation of 60 children participating per school, leads to a calculated total sample size of about 1200 children. The baseline sample included 1103 children from which a subsample of 636 participants (291 boys, 345 girls) between the ages of five and nine years was used that participated in the physical and motor testing. Nine-year-olds were excluded from this study because of low numbers of children in this age group. The mean age of this subsample was 6.9 years and included participants divided by the age in years in the following age groups: *n* = 63 (five years), *n* = 180 (six years), *n* = 209 (seven years) and *n* = 184 (eight years).

Measures. Physical and motor testing consisted of both the KTK and TGMD-2 which included items such asjumping sideward (coordination and agility), standing broad jump (explosive strength), balancing backwards (balance), jumping, catching, kicking, and running (motor skill competences and coordination) (Körperkoordinationstest für Kinder (KTK and Test of Gross Motor Development-TGMD-2 [26]. This study only used two locomotor skills (running and jumping) and two object control skills (catching and kicking) from this testing protocol that were assessed by means of process and product assessments where the following process and product testing protocol were followed: The TGMD-2 evaluates the FMS of children in the age group three-ten years, based on the presence or absence of 3–5 behavioral criteria for 12 skills including object control and locomotor skills [26]. Scores across two trials are then summed to provide a raw score for each skill [26]. An overall validity coefficient of 0.89 is reported with an internal consistency reliability coefficient for the locomotor subtest of (0.85) and for the object control subtest (0.78) [26]. The 12 skills of the TGMD-2 are divided into two sub-tests, namely object control skills (striking a stationary ball, stationary dribbling, catching, kicking, overhand throw, and underhand roll) and locomotor skills (running, galloping, hopping, leaping, and horizontal jump). For the purposes of this study, the following four tests that represent two locomotor skills (running and jumping) and two object control skills (catching and kicking) were included: These four FMS are some of the most foundational FMS that are cornerstones of more sophisticated motor and sport skills to develop. It is also often studied by other studies, allowing comparisons between studies. 

Running—Four criteria were used to assess the running skill: (1) the movement of the arms in opposition to the legs with the elbows bent, (2) a brief period where both feet are off the ground, (3) narrow foot placement (landing on heel or toe: not flat footed), and (4) the non-support leg bent approximately 90 degrees (close to buttocks) when running.

Horizontal jump—The horizontal jump from a standing position is assessed as follows: (1) flexion of both knees with arms extended behind the body during the preparatory phase, (2) arms then forcefully extended forward and upward to reach full extension above the head, and (3) the arms thrust downward during the landing. (4) The take-off and landing on both feet should also be simultaneous.

Catching—Three criteria are used to assess the ability to catch a ball that is tossed underhand. (1) Preparation phase where the hands are in front of the body with the elbows in a flexed position, (2) arms then extend while reaching for the ball as it arrives, and (3) the ball is caught by the hands only.

Kicking—Kicking a stationary ball with the preferred foot is assessed with four criteria: (1) a rapid continuous approach to the ball, (2) an elongated stride or leap immediately prior to contacting the ball, (3) the non-kicking foot placed even with or slightly at the back of the ball and (4) kicking the ball with the instep of the preferred foot (shoelaces) or toe.

After a demonstration by the tester, two attempts of each skill were performed and scored according to specific behavioral for each skill (0 = no mastery, 1 = mastery), and then summed to obtain a competence score. The same testers were used for each test item to ensure the tester validity of the results. The 50th percentile of the age-specific TGMD-2 norms which is displayed in Table 3.3 to 3.5, pp. 17 to 19 of the manual [26], was used as a guideline to indicate average mastery.

Product assessments. A product-oriented assessment evaluates the outcome of a movement, which is typically identified as a quantitative score (e.g., speed, distance, or number of successful attempts). Speed over twenty meters was determined by the 20-m sprint test [34]. The time to complete a 20-m run was assessed by electronic timing gates of the Smart Speed testing device with a precision of 0.01 s (Smartspeed, Fusion equipment). The test reliability is reported to be 0.9 in children aged six to 11 years (Smartspeed, Fusion Sports, Summer Park, Brisbane, Australia [35]. The participant starts from a standing position and after an acoustic signal, start the 20-m run. The time to complete the 20 m sprint test [34] was recorded as the measure for speed and the best of two trials are scored as the result. Catching and kicking accuracy were scored out of five attempts, also using the TGMD-protocol (distances between the tester and the participant in the catching skills and the distance to the kicking target of 1.5 cm wide). The distance jumped in the horizontal jumping test was used as a quantitative measure for jumping and was scored in centimeters [36]. Two trials were allowed, and the best trial was recorded in centimeters. This test was performed on a non-slippery mat designed specifically for horizontal jumping. 

Prior to the testing, all participants performed a standardized 5-min warm-up. Senior researchers and honors students in Kinderkinetics tested the participants after they all received training in testing protocol.

### Statistical Analysis

The “Statistica for Windows version 13.3” (Statsoft, Tulsa, OK, USA, 2020) was used for data analysis. Descriptive statistics (Means M), standard deviations (SD), maximum and minimum values, percentages, and frequency divisions were used to describe developmental tendencies in process and product assessments of running, catching, kicking, and standing long jump in the group and by sex. A one-way ANOVA followed by an Unequal N HSD post hoc comparison analyzed process and product age-related differences in these skills, while independent *t*-testing was used to analyze sex differences. The correlation between the process and product assessments of the four skills and the behavioral criteria of each skill was analyzed by means of non-parametric Spearman rank correlation coefficients (r). The significance of correlations (r) was interpreted by using the following cut-off points where the following R-values were used: r = 0.1 small, 0.3 medium and 0.5 large practical significance [37].

## 3. Results

Six hundred and thirty-six children with a mean age of 6.8 ± 0.97 years (54.2% girls and 45.7% boys), participated in the study. To establish developmental differences in FMS mastery levels, the results, of process and product developmental differences of the group, and within and between age groups are firstly described (Table 1) after which sex differences are presented in Table 2. The behavioral component of each skill is then separately reported for boys and girls in Table 3. The proficiency levels attained in the group, and by age and sex are firstly reported and then also compared to the process mastery norms that are reported for same-aged children in the TGMD-2 manual (Table 4). The TGMD-2 is used worldwide for assessing FMS and is considered as a valid, standard assessment of FMS, therefor the 50th percentile of the age-specific TGMD-2 norms as displayed in Table 4 and in the TGMD-2 manual, was used as a guideline to indicate average mastery. The results displayed in Table 1, Table 2, Table 3 and Table 4 is described as interchanging for each FMS to highlight and compare the developmental state and differences found per age and sex. Finally, the results obtained regarding the second objective of the study that analyzed associations between the process and product assessments of the FMS, are displayed in Table 5.

### 3.1. Process: Developmental Age and Sex Differences 

One-way ANOVA revealed significant differences (*p* < 0.05) in the process and product performance scores of all four FMS skills with increasing age (Table 1). The best mastery in the group was found in kicking (90.3%) and running (90.0%), followed by catching (86.7%) and jumping skills (74.8%, Table 1). Independent t-testing revealed significantly higher mean values and percentages mastery (*p* < 0.01) in boys (91.1%; 94.9%; 76.6%, *p* < 0.05) compared to girls in running, kicking, and jumping (89.1%; 86.5%; 73.1%), with no differences in the process (*p* = 0.8856) and product (*p* = 0.2915) scores of catching skills (Table 2). Boys also showed significantly higher percentages of mastery in the various behavioral criteria of these three skills (Table 3). Sex differences were most pronounced in the kicking skills with boys being superior to girls (Table 2 and Table 3). Boys reflected a 1% and 2% better percentage mastery of the arm action and landing sub-components of jumping respectively. Girls, on the other hand, received the ball better during catching, and in jumping, they showed a 1% higher mastery of the getting ready phase and jumping action (Table 3).

### 3.2. Developmental Age and Sex Differences in Each FMS

A separate analysis of each FMS, considering the mastery, sex differences, and a comparison of the EY group with the TGMD-2 norms, was also performed.

#### 3.2.1. Running

Table 1 displays significant process and product differences in running between the ages of five and eight years (*p* < 0.01). The eight-year-olds displayed the highest mastery (93.5%) and running speed (4.23 s). Five and eight-year-olds found foot placement the most difficult behavioral criteria to master, while six and seven-year-olds displayed the poorest mastery of the arm action (Table 3). Overall, boys reflected significantly better mastery of running than girls (91.1% vs. 89.1%, Table 2). They performed 1% better in the arm action, flight phase, and foot place and 2% better in the execution of the leg action than girls, Table 3.

A higher percentage of mastery of the leg and arm actions (94%; 84%, Table 3) of the five-year-old EY group was found compared to the TGMD-2 norms (82%; 73%, Table 3), although they reflected poorer mastery in both the foot placement and flight phase. The six-year-olds in the EY group displayed better performance percentages in only the leg action sub-component (94%; 88%, Table 3). Execution of the foot placement, flight phase, and arm action effectively were also lower in the EY group compared to the TGMD-2 norms. The EY group performed slightly better than the TGMD norms in the leg action and again performed slightly poorer with foot placement and the flight phase. The difference in the mastery of the seven-year-olds was sizable in the arm action where only 83% of the EY group was successful as opposed to the TGMD-2 norms’ indicating mastery of 90%. The eight-year-olds’ mastery was similar to that of the six and seven-year-olds with a higher mastery of the leg action, but with a lower measure against the TGMD-2 norms in the foot placement, flight phase, and arm action sub-components. The EY group had a 6% higher mastery of the leg action. The lower mastery of the mature foot placement of 7%, is considered as large (Table 4). 

#### 3.2.2. Catching

Catching improved significantly (*p* < 0.05) from five to eight-years (69.5%, 81.5%, 89.2% and 94.7%) with eight-year-olds showing the best mastery (94.7%). The catching of the ball was the most difficult behavioral criteria to master in all age groups (Table 3) with the younger subjects that were also considerably poorer compared to the older subjects (five years—19%; six years—48%; seven years—71%; eight years—86%, Table 3). Both girls and boys reached a percentage mastery of 86.7% (Table 2) and showed the same mastery of the readiness position (100%). Girls, however, showed a 2% better mastery of the receiving of the ball position, while boys showed a 1% better mastery of the catching of the ball (0.99 vs. 0.97; 0.64 vs. 0.63, Table 3). Higher mastery percentages were generally present in the catching skills of all four age groups when compared to the TMGD-2 norms. All five-year-olds could perform the readiness position and 90% could execute the receiving position correctly (Table 4). 

The comparison of the EY group with the TGMD-2 norms, reveals a 16% better mastery of the receiving position and a 17% better readiness position in the EY group. However, the five-year-olds presented with much lower mastery of catching (19%), compared to the TGMD norm of 48% (Table 3). Among the six-year-olds, a higher mastery of the receiving and readiness positions (98%; 100%) compared to the TMGD-2 norms (82%; 85%, Table 4) and a slightly lower mastery of the catch (48%) compared to the TGMD-2 norms (51%) are seen. In both the seven and eight- year-old groups, the EY group displayed better mastery in all three behavioral criteria (catch, receive, and readiness positions) compared to the TGMD-2 norms.

#### 3.2.3. Kicking

Kicking was already well mastered at age five (87.1%) but mastery levels still improved significantly (*p* = 0.04) with increasing age; at six years (89.1%), seven years (90.5%) and eight years (92.4%, Table 1). In the five-year-olds, the run-up to the ball showed the poorest mastery (79%, Table 3), while the kicking action was the most difficult to master in both the six and eight-year-olds (85%; 89%), and the elongated step in the seven-year-olds (88%, Table 3). Across all four age categories, the mastery of the kicking action when compared to the other behavioral criteria, showed the biggest challenge. Boys displayed significantly higher mastering of kicking overall, (*p* < 0.05; 94.9% versus 86.5%, Table 2), as well as in all four behavioral criteria, Table 3.

The largest differences were found in the run-up, elongated step, and in the kicking action (5%, 4%, and 7% differences) of boys and girls (Table 4). Interestingly, five-year-olds in the EY group showed considerably better mastery percentages of all four behavioral criteria when compared with the TGMD-2 norms (Table 4). A noticeably higher level of mastery was evident in the elongated step (28%-TGMD norms and 85% (EY group). The six-year-olds also reflect a higher mastery of the elongated step, run-up, and placement of the non-kicking foot, where the highest mastery skill differences were found in the elongated step (32% TGMD-2 norms; 88% EY group). The execution of the kicking action by the EY group, was, however, 4% lower than the TGMD-2 norms. Both the seven- and eight-year-olds in the EY group reflected lower mastery when compared to the TGMD-2 norms in the kicking action and the non-supporting kicking foot sub-components. Both the seven and eight-year-olds in the EY group had higher mastery than the TGMD-2 norms of the elongated step and run-up with the biggest difference seen in the elongated stepping action.

#### 3.2.4. Jumping

Five-year-olds displayed a non-significantly higher percentage mastery (75.4%) of jumping compared to six and seven-year-olds (70.1%, 74.6%) while eight-year-olds had the highest percentage mastery of the four age groups (79.3%, Table 1). Five-year-olds also performed better in certain behavioral criteria than six and seven-year-olds, specifically in the getting ready and arm action criteria. The jumping action and landing components improved progressively with age. The arm action was the most difficult to fully master in all age groups (Table 3). Boys reached a significantly higher qualitative mastery than girls (*p* < 0.01, 76.6% vs 73.1%, Table 2). Minor differences were also found where girls performed 1% better in the getting ready phase and jumping action, while boys showed better mastery of the arm action and landing (1% and 2% respectively, Table 3). At age five, the EY group showed better mastery of the landing, jumping, arm action, and readiness phase in comparison to the TGMD-2 norms (Table 4). The six-year-olds of the EY group also showed better mastery compared to the TGMD-2 norms, except for the landing action where mastery in the EY group was 63% compared to the TGMD-2 norm of 72%. The seven-year-olds in the EY group showed a higher mastery of the jumping skill, arm action, and readiness phase with a slightly lower mastery of the landing (75%) compared to the TGMD-2 norms (76%, Table 4). Similar results were obtained within the eight-year-old group where 80% of the EY group showed a correct landing, although the percentage was lower in comparison with the TGMD norms (88%, Table 4).

### 3.3. Product: Developmental Age and Sex Differences 

A one-way ANOVA (Table 1) reflects clear and significant improvement with increasing age in running speed, catching, and kicking accuracy, and jumping distance (*p* < 0.05) in the group. Sex differences, analyzed by means of independent t-testing, were also visible in the group, where significantly higher mean values were evident in boys compared to girls in running, jumping, and kicking (*p* < 0.01) although insignificant in catching skills (3.39 vs 3.26; *p* = 0.2915, Table 2). 

### 3.4. Associations between Process and Product Assessments

Table 5 displays the results of the associations between the process and product assessments of the four FMS. Significant correlations coefficients were established between the process and product assessments of each of the four skills in the group, ranging between r = 0.20 (kicking) and r = 0.79 (catching), where catching and jumping skills (r = 0.40) showed the largest associations and kicking skills the smallest associations. Running skills showed a moderate, inverted negative correlation of r = −0.33 in the group. Foot placement revealed the biggest correlation (r = −0.29; and r = −0.28, both trials), while the arm action reflects the smallest correlation between the assessments (r = −0.12 and r = −0.08 respectively). A stronger correlation emerged between the process and product running performance of girls (r = −0.43) compared to boys (r = −0.32). Foot placement correlated the highest (r = −0.32 and r = −0.33) in both trials in girls and in boys with the time to complete the 20 m dash (r = −0.24, and r = −0.23). The arm action of the girls did not correlate with the running action. Correlations were the largest in the eight-year-old group (r = −0.45), and the smallest in the five-year-old group (r = −0.26). Foot placement reflected the strongest correlation in five and seven-year-olds, flight phase in the six-year-olds, and leg action correlated the highest in the eight-year-olds (Table 5). 

A strong correlation reflecting large practical significance was found between the process and product assessments of the catching skill in the group, (r = 0.79), in both genders (boys, r = 0.80, girls, r = 0.78, *p* < 0.05) and in the different age categories where eight-year-olds reflected the highest correlation (r = 0.77) and the five-year-olds the smallest (r = 0.67).

The quality of catching the ball had the highest correlation with the number of successful catches (r = 0.62 and r = 0.71) in both trials (Table 5). Interestingly, the readiness position of hands in both trials did not correlate with the success of catching the ball.

Only a small, although significant correlation (*p* ≤ 0.05) was established between the process and product assessments of the kicking skill (r = 0.21, Table 5). The elongated step correlated (r = 0.19 and r = 0.12) between the assessments during both trails. No significant correlation was found between process and product measures of kicking in boys, while a small to moderate correlation (r = 0.22) emerged in girls where the placement of the non-kicking foot reflected the strongest correlation. Both the five to six-year-old groups reflected significant correlations in the kicking skill, while weaker correlations occurred among the seven- and eight-year-old groups.

A moderate practically significant positive correlation (r = 0.43, *p* < 0.05) emerged between process and product measures of the jumping skill, with the arm action reflecting the strongest correlation (r = 0.28 and r = 0.23 in both trails). In girls (r = 0.44) the association was larger than in boys (r = 0.40). The strongest significant correlation was found between the arm action of the boys (r = 0.31 and r = 0.28, in both trails) and jumping distance, and in the girls with the landing (r = 0.21 and r = 0.25, both trails). The eight-year-olds reflected the strongest significant correlation between the assessments of the jumping skill (r = 0.51). The jumping action showed no correlation with jumping distance in the group, neither among boys and girls nor in the various age categories.

## 4. Discussion

This study firstly analyzed the level of FMS among typical developing children in South Africa, in the age group of five to eight years, to describe the state and age developmental differences in four foundational FMS skills. Two locomotor and object control skills were assessed by using both process and product assessment approaches. This analysis included a comparison with the 50th percentile of the age specific TGMD-2 norms that are used worldwide as a guide toward age-specific FMS mastery levels. We also identified developmental changes across this age group for running, jumping, kicking, and catching.

Considerable evidence suggests a worldwide trend of declining motor competence in children. Our results confirm average mastery of FMS in five-to-eight-year-old children that is comparable with worldwide trends in FMS mastery. Mastery levels that range between average and good in these object control and locomotor skills are suggested from the results. These findings are therefore, slightly in contrast with findings on the mastery percentages of Australian children that were found to be low to moderate [13]. It was, however, evident that with increasing age, the percentage of higher mastery levels of the EY group compared to the TGMD-2 norms, lowered, which is consistent with lowering trends that are reported in other studies. This result implicates a possible lack of opportunities to sustain the quality of FMS mastery that was found in the earlier ages. A higher prevalence of mastery was demonstrated by American children, near 50–80%, for most skills by the age of nine years [26]. These findings suggest that older children are more at risk of not learning FMS to full mastery. Lack of exposure to quality Physical Education in schools and opportunities to be active after school in older children are suggested as possible reasons for these results. In this regard, a national report on the state of Physical Education in South African schools confirmed that the implementation of this subject in schools is severely compromised, especially in schools residing in poorer communities, while also reporting low availability of resources in such schools [38]. It is also stressed that schools are the best environment to provide all children with the necessary opportunities to develop and learn motor skills [39].

Children in the 5 and 6-year-old EY groups not only performed slightly better when compared to the expected percentages of children to achieve the different behavioral criteria of the TGMD-2 but also showed slightly higher mastery in kicking than running. This finding differs slightly when compared to most literature reporting that running reflects the highest mastery of FMS at a young age [18,38,40] A possible explanation is that soccer is a highly popular sport among South Africans [39,41] and playing informal street soccer or games involving soccer balls and exposure to this game at schooling facilities from a young age might have contributed to the high skill levels of kicking through these play opportunities. Children at these early ages are also still allowed more free play at school while they also tend to play more outdoors with balls which could also have contributed to the high levels of kicking proficiency at a young age. Kicking skills, however, also showed mastery comparable with running skills in all age groups, which confirms the cultural effect of environmental influences such as opportunities to improve skills as also reported in a study on nine-year-old South African children [20].

The analysis of sex differences confirmed significantly higher mastery of running, kicking, and jumping skills in boys, in agreement with other studies [14,42,43], although the quality and quantity of the catching skills of boys and girls were similar. These results again concur with studies reported on pre-school and older South African children [18,20,44] and with studies in other countries [23,45]. A possible reason for the high proficiency of catching skills in both sexes could be due to boys in South Africa participating in culturally popular school sports such as rugby and cricket while netball is a popular school sport that girls engage in [41,46].

Our findings based on age differences that were analyzed, furthermore also confirmed a progressive significant improvement in the mastery levels of all four FMS skills with increasing age as assessed with both the process and product assessments. The highest mastery that was found in running (90.0%) and kicking skills (90.3%), and lower levels of mastery in catching (86.7%) and jumping (74.8%), are also in agreement with the expected levels of mastery as reported by another study [26] in similar skills in the same age period. The results also agree with the level of difficulty of mastery of each of these FMS as reported by other studies [14,22,40].

These development patterns which were also confirmed through other research, convey important information regarding skill mastery in locomotor (running and jumping) and object control skills (catching and kicking). These findings, therefore, bring new understanding of the development of individual locomotor and object control skills, classified as FMS, especially running, kicking, jumping, and catching in the time that children are expected to reach mature levels of skill in each of these FMS. The analysis of the development of the specific behavioral criteria of each skill in the different age groups from five to eight years also added value to the understanding of how these skills develop and what is the main technical challenges in the process to obtain full mastery. The analysis of sex differences in the developmental trajectory of these skills added additional insight into biological set differences that need to be taken into consideration when addressing the improvement of these skills. These results should therefore receive attention from the government that strives toward building a healthier nation, and the Department of Education that needs to enforce these goals in schools by giving more attention to the implementation of quality PE to improve FMS through Physical Education and sporting opportunities. Such an undertaking can support health care systems to improve the healthy behavior of children by contributing to the sustaining of foundational skills in young children.

The results regarding the second objective of the study, namely, to determine the association between the outcomes of process and product assessments of FMS, revealed moderate to large significant associations between the assessments of running, catching, and jumping skills (0.33–0.79) and a smaller association in kicking skills (0.20). Associations increased with age in the running (r range = 0.26–0.45), jumping (r range = 0.39–0.51) and catching skills (r range = 0.67–0.79), and were similar, although slightly higher in girls in all three skills. These results concur with the overall associations found in similar skills in recent studies (0.11–0.81) [28,32]. The moderate to high significant associations that were found between the product and process assessments of jumping in our study, agree with the findings of a study by Logan et al. [28] who also report moderate to large correlations between assessments of the hopping, throwing, and jumping (r range = 0.26–0.88). Although associations reported for hopping and throwing skills could not be directly compared with our results, the associations between process and product assessment of jumping skills were found to be moderate in our group (0.43) and across all groups (0.37 to 0.54). Our study, however, did show a similar association at age seven (0.38) with the association (−0.38) reported by another researcher [32]. On the contrary, our findings showed an increase in associations with increasing age in jumping although two different studies [28,32] reported that the strength of the association decreased in jumping skills to age eight in their studies. This decreased association in their study at the age of eight is ascribed to more emphasis in USA schools to improve locomotor skills in the lower school grades [28].

Associations reported for running were similar to associations reported by another study [32] at age six (−0.45 vs −0.39), seven (−0.56 vs −0.32), eight (−0.45 vs −0.43) and in the group (−0.49 vs −0.39). However, in kicking skills, the association lowered with increasing age (0.26–0.14) and was especially weak in the group of boys (0.09) compared to in girls (0.22). This is again well aligned with findings [32] in another study for their group where both studies showed exact associations (−0.21), with the highest association at age six (−0.47 and −0.26) and lowering and insignificant associations at ages seven (−0.13 vs 0.18) and eight years (−0.03 vs 0.14). The slight differences may be due to different product assessments of kicking as it was differentially scored as kick velocity (m/s) and kicking accuracy in the two studies. High mastery percentages from an early age of kicking skills, as seen in the earlier discussion might be the reason for this poorer association that was found. Although speculative, this early proficiency in kicking might point to small variation between well-skilled and less-skilled children as well as a ceiling effect, and subsequently of further improvement in the association between process and product assessments of kicking.

Associations found in catching that revealed the highest associations between process and product assessments of all four skills in each age group could, however, not be compared directly with any other studies. A possible reason for this high association might be grounded in the complexity of this object control skill for children to fully master at these early ages, based on depth perception and timing constraints. The complexity of the skill can also contribute to bigger variation in scores that can contribute to better discrimination of higher and lower abilities and subsequently influence the results. Our results showed the highest associations between process and product measures at older ages which is different from other findings [32] where associations were the highest at younger ages which can be ascribed to contextual differences in the different countries.

Our findings of associations between the performance criteria of each of the FMS and the product assessments, however, revealed mixed results with no clear patterns of associations that emerged with increasing age. In jumping skills, moderate associations were found at age eight between the preparation and landing phases of the skill and jumping distance, while at age five only the landing showed strong associations. At ages six and seven no significant associations emerged between the process and product assessments of jumping. All behavioral criteria of running showed associations with running speed at age eight, which was again, not the case in younger age groups. The association with catching stayed at a high level, indicating large associations, in all age groups. In kicking skills, the stronger associations, were, however, found at age five (r = 0.44 elongated step and foot placement (r = 0.20), but both associations diminished with increasing age to only emerge again at age eight, although now showing poorer associations. It is, however, clear that the product outcomes of all four FMS were influenced by the different behavioral criteria, although by different behavioral criteria at different ages. Their influences also become stronger in some skills, while lowering in others. This again agrees with other studies that also reported changing patterns of associations between process-oriented assessments across skills or ages [17,28,31]. Changes in the strength of correlations that ranged from poor to strong in balancing skills in four to six-year-old South African children are reported [17]. Another study [31], again, reports that associations still exist in children aged 14 years between process and product outcomes although the strength of associations differed between skills and between boys and girls.

Nevertheless, our data confirm moderate to strong associations between product and process measures of FMS that are consistent with other studies in this regard. Continued research, is, however, recommended in this area for a more comprehensive understanding of these changing influences and the mechanisms behind these changing associations of different behavioral criteria. Based on the range of associations that were found, it should though still also be acknowledged, that the two approaches measure somewhat different constructs of motor competence. In agreement, it is reported that these approaches of measurement are unique and therefore not interchangeable, at least in preschool children with a mean age of 4.6 years, therefore more understanding from research inquiry is needed in this field [47]. Improved understanding of assessment outcomes will, however, provide critical information to the scientific community as it relates to how different FMS assessments may provide different types of information relating to motor competence.

On a practical level, this information can improve practitioners’ knowledge of influential changes in behavioral criteria with age, while advancing their understanding of which behavioral criteria showed the highest association with product outcomes at specific ages that should subsequently receive attention for improvement. Our data would seem to suggest that process and product assessments of the four individual FMS that were assessed in this study, are both effective in assessing FMS, but that both assessments should still be used to capture different aspects and levels of FMS and for a more comprehensive understanding of the product outcomes, especially when reasons behind poor performance are sought and intervention of FMS are planned. Our assertions, therefore, are shared by various researchers [27,28,29,30,32,47].

## 5. Strengths and Limitations

This study had limitations that need to be acknowledged. Although a big sample of children formed part of the study, they were selected based on availability which influence the generalization of the results to broader communities. Children from quintile one and two schools which are schools that represent the poorest school status, were not part of the studied group which might have influenced the outcome of the results as poor socio-economic circumstances are associated with poorer motor skills development. The strong point of this study is that a considerable number of children were sampled and tested between five and eight years, and therefore extends previous research that related to this research area in this age group. The additional analysis of the behavioral criteria of the different FMS that were included in the analysis strengthens the understanding and the applicability of the findings. The TGMD-2 norms which are largely based on American children, also enabled comparisons with the mastery of children in other countries since the measuring instrument is used worldwide. The validation of TGMD-2 for South African children is, however, also recommended for future FMS research in the South African population.This is a first study of South African children in the five to eight-year-old group with a relatively larger sample size to determine the state of their FMS as assessed by both product and process measurements, and therefore, adds to the worldwide findings of the mastery but also the current state of FMS proficiency in modern day children.

## 6. Conclusions

Our findings confirm the general improvement of FMS between five and eight years, although it also revealed specific and unique patterns of FMS development that are different from studies conducted in other countries, suggesting unique influences and contributions to FMS development in specific countries that need to be taken into consideration in worldwide comparisons of FMS. The unique developmental patterns that were established in especially kicking skills ropose the feasibility that the mechanism of learning gross motor skills is also attributable to factors other than temporal trends. The influences of the contextual nature and the cultural environment on developmental outcomes of FMS therefore, need continued inquiry from researchers. The extent to which FMS proficiency levels are maintained into later childhood also needs more research emphasis to determine if the school environment is equipped to sustain these skills. Developmental progressions in FMS such as throwing, and stability skills should also be studied to obtain a more holistic picture of overall motor competence in children.

The strength of associations between process and product assessment also appears to vary depending upon the skill and the behavioral criteria that contributed to the association at different ages. Our findings do, however, show that there is still neither a clear nor a comprehensive understanding of the relationship between process- and product-oriented assessments of FMS competence in children and that more research is required in this area. This information can again be used as guidelines for further research related to age-appropriate skills development and the importance of FMS, as well as specific behavioral criteria within a specific skill, at a certain age. The assessment of motor competence is becoming increasingly important in young children and therefore accurate information regarding associations between process and product assessments of motor competence is needed in this scientific field. Additionally, improved awareness by means of education and enhanced knowledge about the relationship between motor competence assessments on health-related motor testing in children will contribute to health promotion in the further course of life.

## Figures and Tables

**Table 1:** Process and product performance of FMS among five to eight-year-olds, per age group.

	Running Process./8	Running Product (s)	Catching Process./6	Catching Product. (Number)	Kicking Process./8	Kicking Product. (Number)	Jumping Process./8	Jumping Product. (cm)
	*GROUP, 5–8 years (n = 636)*							
**Mean**	7.20 *	**4.39**	5.20 *	**3.32**	7.22 *	**2.97**	5.98 *	**111.88**
**SD**	**1.12**	**0.44**	**0.91**	**1.64**	**1.18**	**1.28**	**1.38**	**18.41**
**%**	** 90.0**	** -----**	** 86.7**	**-----**	**90.3**	** -----**	** 74.8**	**-----**
**Min./Max**	** 2–8**	**2.26–6.59**	**0–6**	** 0–5**	**1–8**	**0–5**	** 1–8**	**66.10–190.0**
	* 5 years (n = 63) *							
Mean	6.86	4.86	4.17	1.37	6.97	2.90	6.03	101.84
SD	1.32	0.53	0.98	1.46	1.18	1.38	1.76	13.91
%	85.8	-----	69.5	-----	87.1	-----	75.4	-----
Min.–Max.	3–8	3.12–5.81	2–6	0–5	4–8	0–5	2–8	70.9–145.7
	* 6 years (n = 180) *							
Mean	6.97	4.50	4.89	2.67	7.13	2.75	5.61	106.94
SD	1.28	0.35	0.83	1.61	1.20	1.37	1.46	15.66
%	87.1	-----	81.5	-----	89.1	-----	70.1	-----
Min.–Max.	2–8	3.76–6.10	4–6	0–5	2–8	0–5	1–8	73.40–153.9
	* 7 years (n = 209) *							
Mean	7.26	4.29	5.35	3.70	7.24	2.92	5.98	113.09
SD	0.99	0.39	0.85	1.36	1.16	1.28	1.19	16.91
%	90.8	-----	89.2	-----	90.5	-----	74.6	-----
Min.–Max.	4–8	2.26–5.27	0–6	0–5	1–8	0–5	1–8	75.80–160.2
	* 8 years (n = 184) *							
Mean	7.48	4.23	5.68	4.21	7.39	3.26	6.33	118.86
SD	0.91	0.41	0.60	1.12	1.15	1.11	1.26	20.91
%	93.5	-----	94.7	-----	92.4	-----	79.3	-----
Min.–Max.	3–8	3.40–6.59	4–6	0–5	2–8	0–5	2–8	66.10–190.0

SD = standard deviation; % = percentage mastery; Min = minimum value; Max = maximum value. Note: * Changes from 5 to 8 years were significant in all four skills (*p* < 0.05).

**Table 2:** Process and Product FMS Performance differences in the group of five- to eight-year-olds, by sex.

	Running Process./8	Rnnning Product (s)	Catching Process/6	Caching] Product (Number)	Kicking Process /8	Kicking Product. (Number)	Jumping Process./8	Jumping Product (cm)
	*Boys (n = 291)*							
Mean	7.29	4.26	5.19	3.39	7.60	3.19	6.12	117.00
SD	1.05	0.40	0.92	1.62	0.77	1.17	1.32	19.06
%	91.1	-----	86.7	-----	94.9	-----	76.6	-----
Min.–Max.	3–8	2.26–5.69	2–6	0–5	5–8	0–5	2–8	80.95–165.2
	*Girls (n = 345)*							
Mean	7.13	4.49	5.20	3.26	6.92	2.80	5.85	107.60
SD	1.16	0.45	0.91	1.66	1.35	1.34	1.42	16.71
%	89.1	-----	86.7	-----	86.5	-----	73.1	-----
Min.–Max. *p*-value	2–8 <0.01	3.12–6.59 <0.01	0–6 0.8856	0–5 0.2915	1–8 <0.01	0–5 <0.01	1–8 <0.01	66.10–190.0 <0.01

SD = standard deviation; % = percentage mastery; Min = minimum value; Max = maximum value.

**Table 3:** Descriptive performance criteria of the running, catching, kicking, and jumping skills of boys and girls.

	Boys				Girls			
**Behavioral Criteria**	**N**	**Mean**	**SD**	**Min-Max**	**N**	**Mean**	**SD**	**Min-Max**
	**Running**							
Arm action	286	0.86	0.35	0–1	346	0.85	0.38	0–1
Flight phase	286	0.96	0.20	0–1	346	0.95	0.22	0–1
Foot placement	286	0.88	0.33	0–1	346	0.87	0.34	0–1
Leg action	286	0.94	0.23	0–1	346	0.92	0.25	0–1
	**Catching**							
Readiness position	286	1.00	0.03	0–1	346	1.00	0.08	0–1
Receiving position	286	0.97	0.17	0–1	346	0.99	0.16	0–1
Catching	286	0.64	0.48	0–1	346	0.63	0.48	0–1
	**Kicking**							
Run-up	286	0.95	0.21	0–1	346	0.90	0.35	0–1
Elongated step	286	0.94	0.23	0–1	346	0.90	0.35	0–1
Non-kicking foot pos.	286	0.96	0.20	0–1	346	0.94	0.28	0–1
Kicking action	286	0.94	0.24	0–1	346	0.87	0.38	0–1
	**Jumping**							
Readiness phase	286	0.82	0.38	0–1	346	0.83	0.41	0–1
Arm action	286	0.64	0.48	0–1	346	0.63	0.49	0–1
Jumping action	286	0.85	0.36	0–1	346	0.86	0.37	0–1
Landing	286	0.76	0.43	0–1	346	0.74	0.46	0–1

N = Number, M = Mean, SD = Standard deviation, Min = Mininmum, Max = Maximum.

**Table 4:** Percentage mastery of the behavioral criteria by five- to eight-year-olds compared to TGMD-2 mastery percentages.

Behavioral Criteria	5 yr TGMD (%)	5 yr EY (%)	6 yr TGMD(%)	6 yr EY (%)	7 yr TGMD (%)	7 yr EY (%)	8 yr TGMD (%)	8 yr EY (%)
	**Running**							
Arm action	73	84	89	78	90	83	94	93
Flight phase	97	89	98	93	99	98	99	97
Foot placement	93	77	94	85	94	91	96	89
Leg action	82	94	88	94	90	93	90	96
	**Catching**							
Readiness position	83	100	85	100	93	99	95	100
Receiving position	74	90	82	98	94	99	94	99
Catching	48	19	51	48	68	71	80	86
	**Kicking**							
Run-up	77	79	86	90	91	93	91	92
Elongated step	28	85	32	88	50	88	67	96
Non-kicking foot pos	87	98	90	94	94	92	95	94
Kicking action	84	88	89	85	92	90	93	89
	**Jumping**							
Readiness phase	44	87	75	77	76	79	82	83
Arm action	30	65	43	59	49	59	55	67
Jumping action	74	81	81	82	83	86	84	87
Landing	48	69	72	63	76	75	88	80

% = percentage; EY = ExAMIN Youth SA study, yr = year.

**Table 5:** Associations between the process and product assessments of running, catching, kicking, and jumping skills, per group, sex, and age.

	Group	Boys	Girls	5 Years	6 Years	7 Years	8 Years
	**Running (seconds)**						
Arm action (T1)	−0.12	−0.17	−0.09	−0.02	−0.06	−0.09	−0.19
Arm action (T2)	−0.08	−0.02	−0.09	−0.08	−0.08	−0.08	−0.03
Flight phase (T1)	−0.21	−0.14	−0.28	−0.08	−0.34	−0.14	−0.03
Flight phase (T2)	−0.23	−0.18	−0.25	−0.20	−0.34	−0.13	−0.17
Foot placement (T1)	−0.29	−0.24	−0.32	−0.15	−0.29	−0.25	−0.29
Foot placement (T2)	−0.28	−0.23	−0.33	−0.21	−0.29	−0.23	−0.31
Leg action (T1)	−0.14	−0.11	−0.17	−0.08	−0.09	−0.13	−0.34
Leg action (T2)	−0.18	−0.12	−0.22	−0.16	−0.17	−0.13	−0.30
Running Process Total	−0.33	−0.32	−0.43	−0.26	−0.39	−0.32	−0.45
	** Catching (number) **						
Readiness (T1)	0.03	−0.02	0.06	-	−0.06	0.12	-
Readiness (T2)	0.03	-	0.03	-	-	0.09	-
Receiving (T1)	0.25	0.27	0.23	0.23	0.24	0.22	0.28
Receiving (T2)	0.18	0.18	0.18	0.26	-	0.09	-
Catching (T1)	0.62	0.64	0.60	0.57	0.52	0.50	0.51
Catching (T2)	0.71	0.72	0.71	0.69	0.61	0.62	0.66
Catching Process Total	** 0.79 **	** 0.80 **	** 0.78 **	** 0.67 **	** 0.74 **	** 0.69 **	** 0.77 **
	** Kicking (number) **						
Run-up (T1)	0.06	0.09	0.02	0.08	0.14	0.10	−0.09
Run-up (T2)	0.11	0.02	0.13	0.10	0.14	0.11	0.08
Elongated step (T1)	0.19	0.10	0.20	0.44	0.17	0.13	0.10
Elongated step (T2)	0.12	0.06	0.12	−0.09	0.10	0.13	0.20
Non-kicking foot (T1)	0.10	0.01	0.13	0.12	0.19	0.05	0.11
Non-kicking foot (T2)	0.15	−0.01	0.21	0.27	0.20	0.09	0.15
Kick action (T1)	0.08	0.01	0.08	0.09	−0.01	0.15	0.10
Kick action (T2)	0.06	0.02	0.03	0.07	0.13	−0.02	0.05
Kicking Process Total	** 0.20 **	** 0.09 **	** 0.22 **	** 0.26 **	** 0.26 **	** 0.18 **	** 0.14 **
	** Jumping (distance in cm) **						
Preparation phase (T1)	0.17	0.15	0.18	0.16	0.16	0.14	0.20
Preparation phase (T2)	0.20	0.16	0.23	0.18	0.11	0.22	0.33
Arm action (T1)	0.28	0.31	0.25	0.22	0.30	0.25	0.30
Arm action (T2)	0.23	0.28	0.19	0.13	0.30	0.19	0.27
Jump action (T1)	0.05	−0.01	0.09	0.04	0.06	−0.05	0.09
Jump action (T2)	−0.03	−0.03	−0.02	−0.02	−0.05	0.06	−0.14
Landing (T1)	0.20	0.19	0.21	0.39	0.07	0.18	0.23
Landing (T2)	0.21	0.13	0.25	0.47	0.14	0.05	0.25
Jumping Process Total	** 0.40 **	** 0.40 **	** 0.44 **	** 0.39 **	** 0.35 **	** 0.40 **	** 0.51 **

-—Refers to no variation in the raw data, T1 = trial 1, T2 = trial 2.

## Data Availability

The dataset is the property of the North-West University under supervision of Ruan Kruger. In this regard, R. Kruger should be contacted if, for any reason, the data included in this paper needs to be shared. R. Kruger and M.A. Monyeki are the principal investigators of this study and gave permission that we can use the data.
